# Exploring the Usability and Acceptability of the FoodMATS-Youth App for Monitoring Food Marketing Exposures: Mixed Methods Study

**DOI:** 10.2196/79306

**Published:** 2025-12-09

**Authors:** Idris Opeyemi Bamigbayan, Laurie K Twells, Kirby L J Shannahan, Rachel Prowse

**Affiliations:** 1 Division of Population Health and Applied Health Sciences, Faculty of Medicine, Memorial University of Newfoundland, 39 Clinch Crescent A1B 3V6, St. John's, NL, Canada, 1 709-864-6622; 2 Faculty of Business Administration, Memorial University of Newfoundland, St. John's, NL, Canada

**Keywords:** food, marketing, mobile apps, feasibility studies, data collection

## Abstract

**Background:**

Unhealthy food and beverage marketing influences children’s attitudes, preferences, and behaviors toward food. Most research studies on children’s exposure to food marketing focus on single settings, media, or marketing channels, precluding cumulative estimates of food marketing exposure across children’s daily lives. Therefore, there is a need for tools to measure food marketing across settings.

**Objective:**

This study aimed to test the feasibility of a mobile app to assess food marketing observed by youth aged 13-17 years across settings in their daily life in Newfoundland and Labrador.

**Methods:**

Using a digital app, FoodMATS-Youth (Food and Beverage Marketing Assessment Tool for Settings and Youth; developed by MetricWire Inc), 23 participants photographed food marketing they observed over 3 days. Each participant completed a feedback survey on the usability and acceptability of the digital app assessed through a 5-score rating of feasibility outcomes. They also took part in focus groups, sharing their experiences with the app, and these data were thematically analyzed. Descriptive analyses of app-derived feasibility metrics were also conducted.

**Results:**

The app had high usability and acceptability based on the feasibility outcomes, app-derived feasibility metrics, and focus group responses. For feasibility outcomes, app navigation had the highest rating at 4.7, similar to ease of use and app responsiveness at 4.48; convenience received the lowest rating at 4.0. App-derived feasibility metrics, such as user compliance, response, and app completion rates were also high at 92%, 85.2%, and 92%, respectively. A total of 146 photos of food marketing were submitted by participants through the app. Focus groups showed great participant satisfaction with the app’s interface and functionality.

**Conclusions:**

This study found that the FoodMATS-Youth mobile app is highly feasible for monitoring food marketing exposures across multiple settings (eg, social media and grocery stores) and was well received by our participants. The FoodMATS-Youth has the potential to efficiently improve food marketing research in Canada and internationally and generate data that can inform comprehensive food marketing policies.

## Introduction

Unhealthy food and beverage marketing influences children’s food preferences, attitudes, and consumption [1]. Research shows that children are exposed to marketing through many channels and settings, including websites, apps, social media, school, stores, restaurants, video games, and recreational centers [2]. There have been calls by the World Health Organization to prioritize protecting children from unhealthy food marketing in all settings [3]. Despite the evidence on the impacts of food marketing, such as its influence on the excessive consumption of unhealthy food products, which leads to unhealthy weight gain and diet-related chronic diseases [4], comprehensive monitoring is still needed to inform effective policies restricting food marketing to protect children [5]. Monitoring approaches tend to focus on single settings or marketing channels, with a greater focus on television advertising [6]. These methods are likely to underestimate the level of exposure [6]. With the growth of digital marketing, there is also an increasing interest in assessing food marketing in online media [6,7]. However, current methods and approaches are not adequately designed to capture children’s actual exposure to online food marketing, often measuring only partial or potential exposures [7]. Methods have not been developed to reflect the ubiquitous nature of food marketing or to monitor the transfer of food marketing exposures between settings or media in response to restrictive policies. Methods to comprehensively monitor food marketing exposures experienced by children are needed to fill notable research gaps and adapt to the changing food marketing landscape [8].

Crowdsourcing data from children and parents on their food marketing exposures is a relevant strategy for gathering data for monitoring efforts and can be done using custom mobile apps or websites [6,9,10]. Despite the potential that mobile apps have to be used for crowdsourcing relevant food marketing data [9], only 1 mobile app, GrabFM (Grab Food Marketing), has been used to collect data on food marketing in 2 studies [9,10]. These studies only explored teen-targeted food marketing, potentially underestimating the presence and power of marketing instances that participants are exposed to or influenced by but do not perceive as teen-targeted. Considering this gap, our study aimed to test the use of the FoodMATS-Youth (Food and Beverage Marketing Assessment Tool for Settings and Youth) app to capture all marketing types that youth encounter, not only what they believe targets them. This is particularly important because evidence shows that due to numerous research gaps, overall food marketing exposure is underestimated [11]. Although mobile apps are increasingly being used in research, most studies that use them are intervention studies [12]. Mobile apps have also been successfully used as a data collection tool in diary studies where participants use their smartphones to submit photos and complete tasks, showing the versatility of apps in research [13].

Smartphone ownership has increased significantly over the years, and the growing ownership has been observed in Canada, with 84% of Canadians owning a smartphone for personal use in 2020 [14]. Statistics Canada also reported that 96% of Canadians aged 15‐24 years used a smartphone in 2020 [15]. This results in an increase in mobile app usage. The widespread usage of smartphones makes them an ideal tool for research and health interventions, as they can be used to collect real-time data [16]. However, this could be undermined by limited access to smartphones among marginalized populations or a lack of proficiency in the use of mobile apps [17]. Teenagers use their phones daily and can thus be engaged in research that they find acceptable and in an environment they deem familiar [18]. In addition to being an efficient tool for data collection, mobile apps can also be used to engage with hard-to-reach populations [19]. This is particularly important in Newfoundland and Labrador, as it has a dispersed population, and a mobile app can enable crowdsourcing of food marketing data not only from metro areas but also from small towns as well. Youth in Newfoundland and Labrador have some of the poorest diets in Canada based on school-day dietary quality [20]. Additionally, youth in Newfoundland and Labrador had the highest rates of overweight and obesity (37.5%) among those aged 12‐17 years in 2022, according to the Canadian Community Health Survey [21]. The development of mobile apps and other digital interventions requires feasibility testing [13], which determines if the app is appropriate for the objectives and its audience. Usability and acceptability are common outcomes often assessed in mobile app feasibility studies [22-24]. Usability refers to how much a tool can be used to achieve its goals while maintaining user satisfaction efficiently [25]. Acceptability describes how much the users of an intervention consider it to be appropriate [26]. Some measures often evaluated under usability and acceptability include ease of use, responsiveness, navigation, satisfaction, recommendations, completion, convenience, efficiency, and attractiveness [23,24,27].

This study explored the feasibility of using a mobile app to capture food marketing exposures among youth (aged 13‐17 years). We aimed to assess usability and acceptability through feasibility outcomes, measures of mobile app usage, and the experiences of youth using the app. Based on the Technology Acceptance Model and its modifications [28], perceived ease of use and perceived usefulness are key determinants of technology adoption. As such, usability and acceptability in this study focus on applicable outcomes, such as ease of use, convenience, responsiveness, navigation, willingness to use again, and usefulness of training resources. Usability and acceptability have been found to be essential in the adoption of digital data collection tools and mobile health apps [13,29].

## Methods

### Research Design

This was a convergent mixed methods study that used both qualitative (QUAL) and quantitative (QUAN) tools to assess the feasibility of a mobile app [30]. This study tested the feasibility of a mobile app to assess food and beverage marketing exposures among youth using backend mobile app usage data, feedback surveys, and focus group discussions. The app usage data were used to assess app-based feasibility metrics while the feedback surveys and focus groups were used to assess and understand the usability and acceptability of the mobile app. In this study, both quantitative and qualitative data were collected and analyzed concurrently, then mixed for interpretation to comprehensively assess usability and acceptability. Concerning weighting, the quantitative and qualitative data in this study were given equal weighting, as both were used to assess usability and acceptability. Both data types were integrated when presenting the “Results” and in the “Discussion” sections for interpretation. We have combined qualitative data in exemplar quotes with quantitative results and contextualized feasibility ratings with focus group data in the discussion. Qualitative and quantitative data in this study complement each other and offer a more in-depth understanding of the feasibility outcomes beyond ratings.

### Participant Sampling

Participants were required to be between the ages of 13 and 17 years, live in Newfoundland and Labrador, and have access to a mobile phone for the study duration. Participants were recruited through Community Youth Networks and targeted social media advertisements (Facebook [Meta Platforms, Inc] and Instagram [Meta Platforms, Inc]). The Community Youth Networks are a provincial government-funded initiative providing community-based developmental programs for youth aged 12‐18 years in NL. These networks contributed to participant recruitment by sharing the study information with their members. Facebook and Instagram advertisements were used to enroll older youth (aged 15‐17 years), as we were unable to sufficiently recruit that age group through the Community Youth Networks. Parental consent and child assent were provided for participants aged 13‐14 years, while 15‐17-year-olds provided their consent.

An initial sample size of 24 participants was chosen based on similar photo-based studies in Canada [31,32]. In addition, this is a pilot study, and a sample of 24 participants was deemed sufficient to achieve the purpose of the study. We attained data saturation with 23 participants. At the end of the study, each participant was offered a $30 (CAD $1=US $0.75) electronic gift card as a thank-you for their time and effort throughout the study, regardless of whether all study activities were completed.

### FoodMATS-Youth App

**Figure 1.**
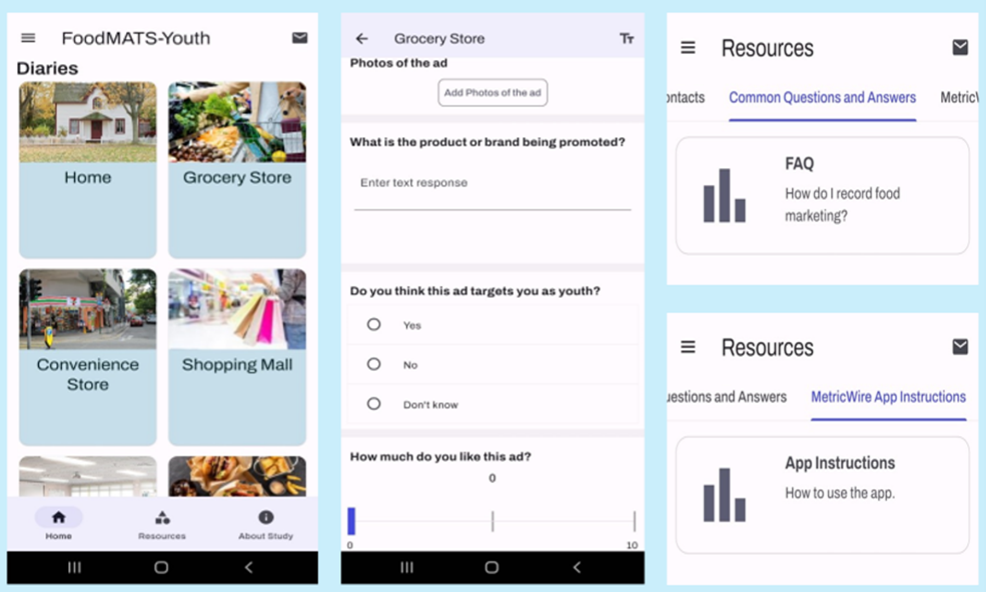
FoodMATS-Youth (Food and Beverage Marketing Assessment Tool for Settings and Youth) mobile app showing the home, data collection diaries, and resources (left to right).

**Figure 2.**
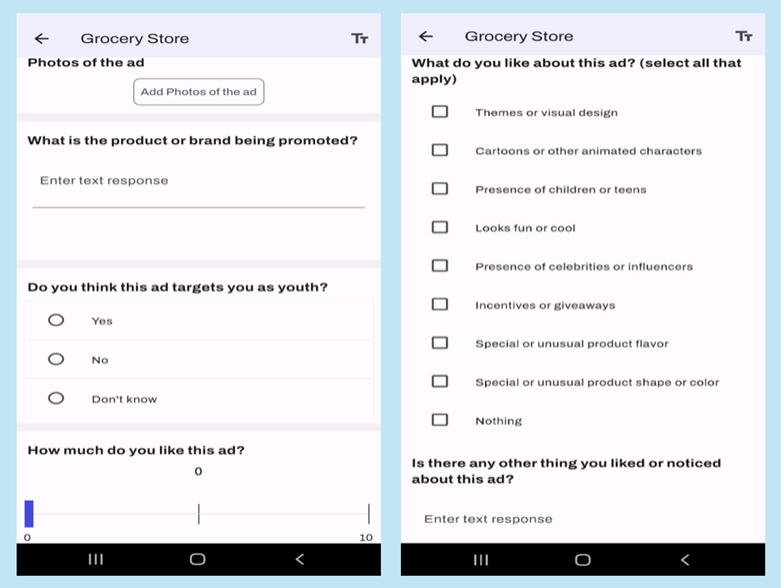
Questions and prompts in the FoodMATS-Youth (Food and Beverage Marketing Assessment Tool for Settings and Youth) about the marketing features of the advertisement.

We collected data using our app, FoodMATS-Youth, which was created on the Catalyst by Metricwire application, a Canadian software application company with a record of facilitating digital health research. The FoodMATS-Youth had 3 main pages, namely “Home,” “Resources,” and “Data Collection Diaries” (Figure 1). It also had an “About Study” page, a “Consent Information” page at the beginning of the study, and a “Feedback Survey” page at the end. The home menu contained diaries for 13 settings or media (home, grocery store, convenience store, shopping mall, school, fast-food restaurants, sit-down restaurants, youth center, recreation and sports center, movie theater, public transportation, social media, and other), and each location had a cover image representative of it (Figure 1). The settings were selected based on locations that youth often visit and existing literature [33]. There was also a setting called “other” where participants could specify the location of the food marketing. The diaries contained instructions, an option to upload or take photos, and a series of questions on the marketing features of the advertisement as shown in Figure 2. These questions were informed by Health Canada’s indicators for monitoring the message, design, and techniques of advertisement or marketing instance (Health Canada, unpublished data, October 2022). The mobile app included push notifications to remind participants to make diary entries when they see food marketing, and these notifications were delivered 6 random times per day, with a minimum interval of 2 hours apart and between 9 AM and 9 PM. The notifications read, “Where are you? Don’t forget to take pictures of food ads you see!” The app also provides instant messaging for on-demand help from the research team if issues arise. The mobile app was developed to work on Android and iOS devices and offers real-time tracking of submissions and app usage.

### Data Collection

Participants were required to provide informed consent through the Qualtrics (Qualtrics International Inc) survey tool after reading the consent form. Consenting individuals provided demographics through a Qualtrics survey, including names, phone numbers, and emails of participants and parents, and smartphone access. Next, participants were trained on how to use the mobile app in a virtual training session via Webex by Cisco software. Multiple training sessions were held based on participants’ availability and preferences, and all participants were required to attend at least one training session. Participants were trained using a mobile app user guide detailing a step-by-step process of using the app. Training resources were sent to each participant alongside the app invite link after they had completed the training. They then downloaded the Catalyst by MetricWire application via their respective App Stores or Play Stores and used the FoodMATS-Youth app to record food marketing in their lives. In the FoodMATS-Youth, they completed personalized diary entries each time they noticed food marketing over 3 days (2 weekdays and 1 weekend) by uploading photos of food marketing instances and answering a series of questions on the marketing features. Mobile app–derived outcomes, including study recruitment and retention, mobile app response and completion rate, response pattern, compliance rate, breadth of mediums, quality of photos, and app language were collected from backend mobile app usage data.

After the study period, participants completed a feedback survey on the usability and acceptability of the app, which was embedded in the FoodMATS-Youth app. This survey was used to assess the feasibility outcomes (ease of use, convenience, responsiveness, and navigation of the mobile app; the effectiveness of the training resources; and their willingness to use the mobile app again). Participants were asked to rate each of these factors with a score between 1 and 5, with a score of 5 being the best.

Finally, all participants were invited to attend focus groups organized by age (13‐14 years; 15‐17 years) to explore their experiences with the app. We made use of a focus group guide (Multimedia Appendix 1). The focus groups were conducted online via Webex, a videoconferencing platform, as this enables ease of participation for participants living outside the city. The focus groups were recorded on Webex and transcribed verbatim afterward. The data collection period for this study was from June 2023 to April 2024. The study timeline for each participant from recruitment to the focus group was based on their availability and preferences. Training sessions were available as the first batch of recruitment was in progress, and recruited participants began data collection a day or two after attending a training session. Participants were required to register on the app, take photos of food marketing exposures, and complete the feedback surveys within 7 days.

Since participants were required to self-report marketing exposures, there was a risk of self-report bias and data unreliability. To address this, all participants attended a virtual training session where they were instructed on how to accurately identify and submit food marketing exposures. To help ensure that photo submissions reflected actual exposures, participants were informed that they would receive their incentives regardless of whether they completed the study. To minimize incomplete submissions, participants were informed whenever a submission did not include a photo. Additionally, to reduce missing data, participants were encouraged to upload every instance of food marketing they encountered, even if they were unsure whether it qualified as marketing.

### Data Analysis

The quantitative and qualitative data from this study were analyzed using SPSS (version 29; IBM Corp) and NVivo 14 (Lumivero), respectively. Analyses compared feasibility outcomes by age group (13‐14-year-olds; 15‐17-year-olds). Mobile app–derived outcomes assessed included recruitment and retention rate, response rate, completion rate, response pattern, compliance rate, breadth of mediums, and quality of photos. Descriptive analyses were conducted, including measures of central tendency (mean and median) for continuous variables. Cross-tabulations were conducted using Fisher exact test for categorical variables due to small cell counts.

Specifically, the frequency of photo submissions was calculated according to settings or media and the proportion of settings or media that had submissions was analyzed according to age group. The mean rating for each feasibility outcome was calculated according to age group. The distribution of submissions overall and according to age group was also illustrated using a boxplot with the median and IQR. Cross-tabulations using Fisher exact test were calculated to assess whether the rating category for feasibility outcomes differed by age group. The rating categories used for the Fisher exact test were low (≤3) and high (4-5).

We conducted a thematic analysis [34] of focus group data to assess feedback on experiences while using the mobile app to understand usability and acceptability better. To conduct the thematic analysis, we first transcribed the focus groups and reviewed the transcripts multiple times while taking note of initial ideas and patterns. We then coded the responses inductively, identifying all meaningful concepts. These codes were data-driven and included all aspects of the data that related to the usability and acceptability of the mobile app. After coding and collating all the codes, we sorted them into identified themes by combining related codes into an overarching theme. We created a thematic chart showing themes, subthemes, and related codes. The themes were then reviewed for internal homogeneity and external heterogeneity. The final themes were then named and defined, after which they were synthesized and presented [34]. Verification strategies were used in this study to ensure rigor, consistency, and suitability across the research process, including methodological coherence, memoing, negative case analysis, concurrent data collection and analysis, and sampling sufficiency. To ensure methodological coherence, the methods used in data collection and analysis were carefully selected to achieve the objectives of this research [35,36]. Memoing of themes and ideas was carried out during and after data collection, which helped identify if the research objectives were being answered [37]. The negative cases, which are those that contradict emerging patterns, were explored to assess the deviation from emerging themes [35]. Data collection and analyses were conducted concurrently to identify themes emerging from the focus group and explore them in other focus groups. To achieve sampling sufficiency, we planned to recruit 24 youth across the province. After completing three focus groups, we recognized the need to complete more focus groups to achieve our aim of data saturation, as new information was still being generated. By the eighth focus group, there was no new information, and the study had reached data saturation [35].

### Ethical Considerations

Ethics approval was obtained from the provincial Health Research Ethics Authority of Newfoundland and Labrador (NL) (#20231737). Informed consent was obtained from all participants. Participants aged 13-14 provided assent and parental consent, while those aged 15-17 provided consent. Participation was voluntary and participants could withdraw at any time. Participant privacy and confidentiality were protected in this study. The study data was deidentified during analysis and no identifiable information is included in this paper. There were no significant risks related to this study. At the end of the data collection, each participant was offered a CAD $30 (CAD $1=US $0.75) incentive for their time.

## Results

### Participant Characteristics

A total of 68 youth consented or assented to participate in the study and were invited to participate in the training session. Among them, 39.7% (27) attended the mandatory training session and were eligible to continue participating in the rest of the study. Of those, 22/27 (81.5%) participants completed the study. Half (11/22, 50%) of these participants were 13‐14 years old, and a larger proportion (15/22, 68.2%) were females.

The results are presented below and include app-derived feasibility metrics and the feedback survey feasibility outcomes for 23 participants who used the mobile app and completed the feedback survey. The focus group themes were generated from 22 participants who completed all study tasks, including the focus groups.

### App-Derived Feasibility Metrics

#### Study recruitment and retention

**Figure 3.**
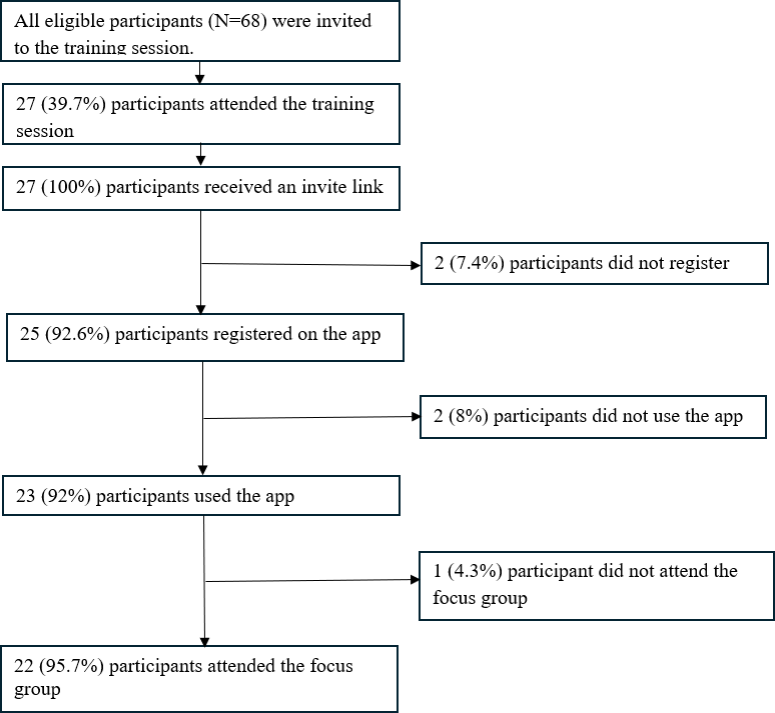
Flowchart showing recruitment and retention of study sample.

In total, 68 participants completed the consent form available through Qualtrics, and about two-fifths (27/68, 39.7%) of these participants attended the training. Of the 41 participants who did not attend the training, 32 (78%) were 15‐17 years old. This disproportionate difference could be attributed to the fact that only older youth were recruited through social media advertisements, resulting in more eligible 15‐17-year-olds overall. A few participants (n=5) dropped out after the training; however, almost all participants who used the mobile app also participated in a focus group (95.7%, 22/23; Figure 3). Participants who dropped out at various stages of the study did so due to time constraints, travel plans, or no communication.

#### Mobile App Response and Completion Rate

For response rate, most participants (23/27, 85.2%) who received the app invite link downloaded the mobile app and used it. Almost all of the participants (23/25, 92%) who registered on the app used it until the end of the study, resulting in a high app completion rate.

#### Response Pattern

**Figure 4.**
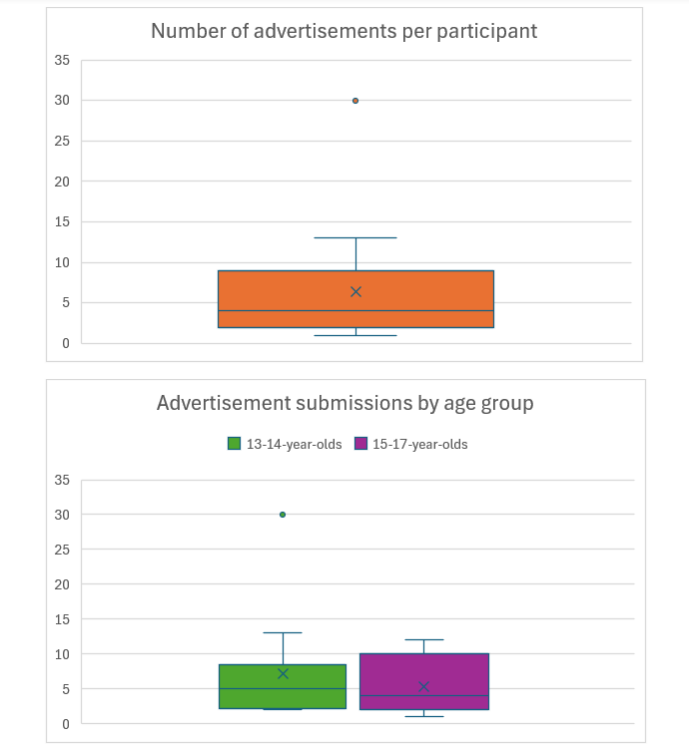
Distribution of total submissions and submissions by age group (x is the mean).

A total of 146 pictures were submitted, with some differences across age groups (Figure 4), with a mean of 6.3 (SD 6.14) pictures per participant and median of 4 (IQR 2-9 ). The number of submissions ranged from 1 to 13 advertisements, apart from 1 participant who submitted 30 advertisements. There were 87 (59.6%) submissions from 13‐14-year-olds and 59 (40.4%) submissions from 15‐17-year-olds. The time of diary submission varied across responses, with about half of the submissions being made from 7 PM to 10 PM.

#### Compliance Rate

Of all the participants who used the app, most (18/23, 78.3%) completed all tasks on the mobile app (app enrollment to feedback survey completion) within the 7-day window. Five participants took up to 15 days to complete these tasks (mean 11.8, SD 2.9; median 12, IQR 9-15).

#### Number of Food Marketing Photos by Settings or Media

The highest number of submissions was from social media, grocery stores, fast food restaurants, and shopping malls (Table 1). Youth aged 13‐14 years tended to have more submissions across a wider variety of settings. Social media recorded the highest number of submissions from both groups.

**Table 1.** Food marketing submissions by setting and age group.

Settings or media of FoodMATS-Youth^a^ App	Submissions (% of total)	Age group (years)	
		13-14, n (% of setting)	15-17, n (% of setting)
Social media	67 (45.9)	36 (53.7)	31 (46.3)
Grocery store	25 (17.1)	18 (72)	7 (28)
Fast-food restaurants	15 (10.3)	8 (53.3)	7 (46.7)
Shopping mall	10 (6.8)	9 (90)	1 (10)
Convenience store	9 (6.2)	3 (33.3)	6 (66.7)
Other^b^	9 (6.2)	7 (77.8)	2 (22.2)
Home (TV^c^, social media, and flyer)	6 (4.1)	3 (50)	3 (50)
Recreation and sports center	2 (1.4)	2 (100)	0 (0)
Movie theater	1 (0.7)	0 (0)	1 (100)
School	1 (0.7)	0 (0)	1 (100)
Public transportation	1 (0.7)	1 (100)	0 (0)
Sit-down restaurants	0 (0)	0 (0)	0 (0)
Youth center	0 (0)	0 (0)	0 (0)
Total	146	87 (59.6)	59 (40.4)

a FoodMATS-Youth: Food and Beverage Marketing Assessment Tool for Settings and Youth

b Other locations were specified by participants in the submissions and included gas station, bowling alley, roadside, hotel, and craft fair.

c TV: television

#### Type of Device and App Language

About two thirds of participants used an iOS device (15/23, 65.2%), and the rest used an Android device (iOS=15 and Android=8). Almost all participants agreed that the language in the mobile app was clear (22/23, 95.7%).

#### Quality of Photos

The quality of the photos submitted was high, showing the product being advertised and the features of the advertisement clearly, which participants mentioned they made sure to do while using the app. Some participants mentioned in the focus group that they did not upload unclear photos they took. The size of photos ranged from 31 kBs to 8.016 MBs. The 15 kB was a picture of a billboard, most likely taken at a distance, and this was one of the poorest photos received. Although this photo had low resolution, features in the advertising were still visible.

### Feasibility (Usability and Acceptability)

#### Average Score Rating for Each Feasibility Question

The average ratings for the feasibility questions ranged from 4.0 (SD 1.04) to 4.7 (SD 0.56), out of a possible average score rating of 5. The feasibility outcome with the lowest overall rating and the lowest rating within each age group was the “convenience” of completing entries. The average rating for each feasibility outcome was slightly higher among 15‐17-year-olds, except for the question on app responsiveness (Table 2).

Focus group data explain average scores in feasibility outcomes. Exemplar quotes by feasibility outcome are included in Table 2. Further description related to ease, convenience, responsiveness, navigation, and willingness to use again is reviewed across three themes on participants’ experiences with the app: (1) influence of app interface and functionality on ease of use, (2) influence of app features on user satisfaction, and (3) practicality and integration of app into daily life.

**Table 2.** Mean ratings for feasibility questions and frequency of each rating according to age group.

Usability and acceptability question^a^ and age group (years)	Average rating^b^, mean (SD)	Ratings				Exemplar quotes from focus groups
		2 (n)	3 (n)	4 (n)	5 (n)	
Ease: How easy was it to use the app?						“I thought that the app’s simplistic design was really helpful and it made it so that understanding what to do was really easy. So, I didn’t have any problems with using the application. It was really like just straightforward. I enjoyed that” (Participant 8, 14 years).
13-17	4.48 (0.79)					
13-14	4.33 (0.98)	1	1	3	7	
15-17	4.64 (0.50)	0	0	4	7	
Usefulness: How helpful was the training resource provided on using the app?						“.....I found it very simple right away actually, and your instructions were very straightforward as well to use the app.” (Participant 21, 16 years).
13-17	4.61 (0.50)					
13-14	4.58 (0.51)	0	0	5	7	
15-17	4.64 (0.50)	0	0	4	7	
Convenience: How convenient was completing mobile diary entries?						“Well, when I was at home, and I was taking screenshots and everything, I would just send them in right away. But when I was out, and I took a few pictures, I was with friends and stuff. So, I kind of just snapped the picture, put my phone away, and was like, I’ll do that when I get home. And that’s what I did.” (Participant 17, 17 years).
13-17	4.0 (1.04)					
13-14	3.83 (1.11)	2	2	4	4	
15-17	4.18 (0.98)	1	1	4	5	
Responsiveness: How responsive was the app?						“It was mostly just after I submitted my answers, it would take a good few seconds to load back into the homepage. So I was afraid if I closed it too fast, it wouldn’t save.” (Participant 22, 15 years).
13-17	4.48 (0.79)					
13-14	4.58 (0.67)	0	1	3	8	
15-17	4.36 (0.92)	1	0	4	6	
Navigation: As it relates to navigation, how easy was it to move in and out of the app?						“I found it very straightforward to use. Once I opened the app, every, I guess, location that I could have found ads in was there, and all I had to do was click it. And the questions were also very straightforward, and I felt the questions were about the point right away. So it was very easy to put in my information and then click submit with and without internet.” (Participant 21, 16 years).
13-17	4.70 (0.56)					
13-14	4.67 (0.49)	0	0	4	8	
15-17	4.73 (0.65)	0	1	1	9	
Willingness to use again: How willing are you to use this app again?						“I don’t think there’s anything that I would change about the app really, because like, I think that like the layout is really nice. And the questions like they said, they were very easy and brief.” (Participant 6, 15 years).
13-17	4.26 (1.01)					
13-14	4.0 (1.13)	2	1	4	5	
15-17	4.55 (0.82)	0	2	1	8	
Total score (out of 30)^c^						
13-17	26.53					
13-14	25.99					
15-17	27.1					

a Rating for each feasibility question ranged from 1‐5. There was no rating of 1 from participants on any of the questions.

b Total possible rating for each question is 5.0.

c This was calculated by adding up the total possible rating for all feasibility questions.

#### Association between age group and rating category

There was no significant association between the proportion of individuals by age group that ranked each feasibilty variable as high or low (2-tailed, P>.05; Table 3).

**Table 3.** Association between age group and rating category.

Feasibility outcomes^a^ and rating category^b^		Age group (years)		*P* value^c^
		13-14, n (%)	15-17, n (%)	
Ease				
Low (<3)		2 (16.7)	0 (0)	.48
High (4-5)		10 (83.3)	11 (100)	
Convenience				
Low (<3)		4 (33.3)	2 (18.2)	.64
High (4-5)		8 (66.7)	9 (81.8)	
Responsiveness				
Low (<3)		1 (8.3)	1 (9.1)	≥.99
High (4-5)		11 (91.7)	10 (90.9)	
Navigation				
Low (<3)		0 (0)	1 (9.1)	.48
High (4-5)		12 (100)	10 (90.9)	
Willingness to use it again				
Low (<3)		3 (25)	2 (18.2)	≥.99
High (4-5)		9 (75)	9 (81.8)	

a The usefulness of training resources could not be computed as all participants rated it high.

b Feasibility scores (1–5) were categorized as low (1–3) and high (4–5) for analysis.

c Fisher exact test was used due to the small sample size.

#### Focus Group Themes

##### Theme 1: Influence of App Interface and Functionality on Ease of Use

Overall, most participants found the application easy to use. This description of the application was often associated with how the design of the application was user-friendly, simple, and streamlined. Most participants were able to onboard and begin using the app easily. A participant said:

*I thought it was very like straightforward to use like, I didn't find it complicated or anything, and I wasn't really like confused about how to use it. Um, yeah, it was it was straight forward, it was easy to use*.

[Participant 17, 17 years]

Participants also expressed that the mobile app made the process of submitting photos brief and efficient. It was quick and easy for participants to find their way through the app when they needed to upload photos and complete the diary entries. Another participant said:

*I found it very straightforward to use. Once I opened the app, every, I guess, location that I could have found ads in was there, and all I had to do was click it. And the questions were also very straightforward, and I felt the questions were about the point right away. So it was very easy to put in my information and then click submit with and without internet*.

[Participant 21, 16 years]

Problems experienced were lagging at the point of submission, leaving them unsure if the submission had gone through. The mobile app did not have a repository where participants could view their previous submissions, so this further influenced the doubt of whether submissions had gone through. There were also a few occurrences where participants completed a submission, but their photos were not attached. One participant recalled:

*It was mostly just after I submitted my answers, it would take a good few seconds to load back into the homepage. So I was afraid if I closed it too fast, it wouldn't save*.

[Participant 22, 15 years]

##### Theme 2: Influence of App Features on User Satisfaction

Users reported different reactions to the features of the FoodMATS-Youth, including prompted questions on the diaries, notifications, cover photos, and locations. Some participants expressed confidence that the questions were relevant and of the right number, as they helped describe the advertisement without the need to put in too much information. Other participants perceived the process of completing questions for every submission as time-consuming. These participants preferred fewer questions or an option to only answer questions on their favorite advertisements. Concerning the app settings, participants reported that the FoodMATS-Youth diary options contained most of the locations they often visited, which made it evident that it was targeted toward their age group. This also emerged as one of their favorite things about the app. The only other location that participants would like to have added to the app was the “roadside.” One participant said:

*Personally, for me, I just like how many options there were. Like, there were a lot of common categories that I go to often, and I also really like there was like an option called others, so you could select that, so it’s not like, you know, “I took this photo, and this category isn't there, so I guess it’s not good.” So I like that that category was included*.

[Participant 9, 13 years]

The cover photos made the home page visually appealing, and they suggested the diaries themselves needed to be more fun and colorful to fit with the home page.

All participants found the push notifications to be helpful reminders, but some also found their frequency annoying, recalling that they came in too often and they no longer needed that many notifications once they got into the habit of uploading pictures. One participant also mentioned how it would be better for the notifications to say different things. She said:

*You know, to be very honest, they're kind of repetitive. You know, they keep saying the same thing over and over, and that’s the thing I don't like about apps. Notifications, I mean, sorry…See, it just told me one ‘where are you’ and that’s the same one as before. Like say, for example, number two will be like. ‘Did you forget to take a picture? Take your picture now,’ or another one can be like, ‘Oh, are you are you eating at a restaurant?’ or something like that*.

[Participant 20, 16 years]

##### Theme 3: Practicality and Integration of App Into Daily Life

Most participants mentioned that the app was minimally disruptive to their daily lives, and they did not find it burdensome. This was sometimes a result of being on break from school or living in small communities with minimal advertising. The simplicity of the submission process was also a factor. Some participants said:

*It wasn't really that difficult. It only takes, like, thirty seconds to take a picture and send it, so it’s not all that hard*.

[Participant 10, 13 years]

It was a pretty simple process, so it didn't really interfere with anything, and I was also on Easter break so that also made it easier

[Participant 15, 17 years]

When asked if they made diary entries immediately upon seeing advertisements, some participants said they did not. They found it more convenient to complete the mobile diaries at home or upload all pictures at the end of the day. A participant said she had fixed goals when shopping and did not pay attention to advertising outside. There were also mixed reactions to taking photos in public as some participants were confident and had no problems taking pictures, while others were nervous. This was not age-specific. Overall, most participants uploaded screenshots from digital media immediately, while other pictures were uploaded from home. Some participants recalled:

*I felt kind of nervous because a lot of the times I thought that other people would think I was taking pictures of them and not like the product or like the advertising. So I was a bit, like, awkward while doing it, um, so I didn't want anybody to think. I was, like, taking pictures of them.*

[Participant 8, 14 years]

Another said:

Yeah. I, felt kind of the same, where it’s kind of awkward to take pictures of advertisement and then punch in some things on your phone. So, it’s definitely easier to take a picture and then fill in the details later.

[Participant 7, 13 years]

## Discussion

### Principal Findings

This study aimed to develop and test the feasibility of the FoodMATS-Youth app as a tool to crowdsource data from youth. The results from the feedback survey and focus groups demonstrate that the FoodMATS-Youth had high usability and acceptability, making it a feasible app to capture food marketing messages observed by youth in their daily lives. This was supported by the high response, completion, and compliance rates during the study. Overall, participants were satisfied with the ease of use, navigation, aesthetics, responsiveness, and appropriateness of the mobile app, and this did not differ by age group. However, there were also challenges faced by participants while trying to make submissions through the app and recommendations by the participants on how to improve it.

Most feasibility outcomes being measured were rated high by participants, indicating excellent usability and acceptability of the mobile app. This is comparable to findings from another study, which evaluated a mobile app’s usability in terms of ease of use, willingness to use again, and clarity [38]. Although there were only 9 participants in that study, 100% (9/9) rated the app as easy to use, and 78% (7/9) expressed willingness to use it again. Similarly, in our own study, 91.3% (21/23) of participants rated the app’s ease of use as high, and 78.3% (18/23) of participants gave a high rating for their willingness to use it again. Mobile app usability often depends on the interface design, which influences ease of use and user-friendliness [39]. This explains why users frequently reported that our mobile app’s simplistic and efficient design was one of the reasons it was easy to use. Previous researchers have reported that adolescents are interested in apps based on their appearance and how they feel, especially if they are aesthetically pleasing and simple to use [40]. The acceptability of the mobile app was generally high, as participants had positive experiences using it and were willing to use it again. They also believed that the app was designed for their age group based on the diaries.

Overall, the convenience of diary completion was satisfactory. However, it had the lowest mean score of all acceptability outcomes measured. Participants stated they had no problems incorporating the app into their daily routine; however, most participants did not complete the diary submissions immediately after taking the pictures. This was not a problem when they submitted photos, as they could refer to the photos to respond to questions about the product being advertised, the setting, and the marketing features present in the advertisement. However, not submitting them immediately meant participants occasionally forgot to upload some photos, which has important implications on the accuracy of monitoring food marketing through digital apps. Convenience is an important factor for mobile app users, and they prefer data collection tools that require minimal effort. If participants need to complete manual entries, like in the case of our app, it should be as convenient as possible. Lack of convenience would limit participant engagement [41] and may affect data quality.

A large portion of the photos submitted was from social media. Many reasons could influence this, including the possibility of participants not visiting as many physical settings during the 3 days of data collection or being preoccupied with other activities while those settings. Some study participants reported that they drove by billboards and did not get an opportunity to take good photos. Looking at findings from the literature, food marketing is significantly moving into the digital space [42,43], which could explain the volume of advertisements from social media. Considering the level of food marketing exposure through social media and the influence it has, it is important to focus food marketing monitoring efforts on this medium [43]. These findings suggest that social and digital media may be a significant source of food marketing exposure to youth, potentially influencing their food consumption patterns.

The mobile app completion rate was very high at 92%. Most of the participants who registered on the app completed the study. This was higher than other feasibility studies testing mobile apps among youth, which had overall completion rates of 33% and 68% [44,45]. However, these studies were interventions and often required a longer app usage period. This could account for the lower completion rates in these studies; unlike ours, where participants had to use the app for 3 days, these studies required usage for 6 weeks. Most of the participants who attended our training session used the app till the end, and they found the training session very useful, as it had one of the highest ratings on the feedback survey. A similar mobile diary study mentioned that they set up meetings with participants for an app demonstration before starting the study to increase participant retention [13]. This could explain our high response and completion rates, as participants already understood how the app worked and had resources to consult when necessary. This suggests that training and demonstrations improve user engagement, thereby contributing to high completion rates among app users.

The push notifications garnered mixed reactions from our participants, and this finding is similar to another study, which found a divide among participants on receiving notifications in general [30]. Another study that used a similar number of notifications daily also reported that participants complained of the notifications being too much, repetitive, and random [46]. This suggests that fewer notifications might be more acceptable. It could also be relevant to consider event-based notifications where the app detects movement using mobile sensors and sends notifications to participants when they leave home [47]. This has to be adequately considered, as participants can feel monitored when they receive notifications [46]. Notifications are essential, as they help remind participants to take photos and could be sent individually to participants who need them more [13]. Notifications on the FoodMATS-youth may also be important in reducing potential underestimation of food marketing exposures.

Due to the app’s self-report design, confirmation bias could have made some participants more likely to notice and report marketing instances that align with their existing beliefs or expectations about unhealthy food marketing, therefore underestimating exposure. However, food marketing is ubiquitous, and the FoodMATS-Youth in its current form could provide information on the most impactful food marketing to our participants, which may have important policy implications. To improve this, the app could be further designed to include automated screen capturing of digital food marketing [48] or video recording using wearable cameras [49]. The findings from this study show that the mobile app has the potential to contribute to Health Canada’s Food and Beverage Marketing Monitoring Framework for Canada [50] through crowdsourcing marketing instances from youth. This proves important not only because it is a digital data collection tool but also because it covers all channels and settings within the scope of the monitoring framework. It also focuses on the population of interest (13 years and older) [50] and can be used to assess the exposure and power of food marketing. While it is being tested in Newfoundland and Labrador, which is one of the locations of monitoring, it could also be further adapted throughout Canada. However, before broader implementation can be suggested, the utility of the mobile app needs to be further explored in larger-scale studies. The app could also be paired with other tools, such as eye tracking, screen capture, or video recording to reduce the risk of self-report bias and improve measurement of food marketing exposure. Ultimately, using the FoodMATS-Youth within Health Canada’s framework could generate data that could be used to advocate for policies restricting food marketing, beyond television and digital media, which are currently the focus of policy efforts [51].

Overall, participants said they enjoyed the mobile app and gave recommendations to improve the experience. Some of the recommendations from the focus group included building transitions or animations to the diaries so the aesthetics can match the home page and make it more visually appealing. Another was alternating the content of the push notifications, so it does not seem redundant. Finally, participants suggested a repository where they can see past submissions, which would be helpful. These are recommendations to be considered in future updates for this mobile app and future app-based studies. The feedback from users suggests that they significantly interacted with the app and shows its usability and acceptability among youth.

### Strengths and Limitations

This was a mixed methods study, which quantitatively assessed the feasibility of the mobile app while exploring youth’s perspectives and experiences with the rigor of qualitative research. This study developed a novel digital app-based data collection tool that can be used to collect data on cumulative food marketing seen by youth. This can include hard-to-reach populations in future studies on food marketing to youth in Canada and beyond. It also has the potential to be integrated with dietary assessment to evaluate relationships between cumulative cross-setting food marketing exposures and dietary intake. This study also adds to the literature on the feasibility of mobile apps as data collection tools. The sample size in this study was small, which has reduced statistical power and may have hidden any significant differences in feasibility outcomes between age groups. While this sample size was sufficient to reach data saturation for qualitative analysis, it was insufficient for more detailed and complex quantitative analysis. The sample size also limits the generalizability of the findings to the general population of Newfoundland and Labrador and of Canada. Also, participants were only required to take pictures for 3 days, and some additional measures may be needed to support participant engagement over a longer data collection period. Some participants did not upload all the pictures of marketing that they took, as they did not always upload the pictures immediately. This likely underestimates the amount of marketing they are exposed to, but it shows those that are potentially more important to youth. This could also be due to attentional bias. Participants sometimes found it more convenient to upload photos at home or at the end of the day. This research still needs to be replicated in a larger sample to investigate differences in app usage by demographic group and ascertain that it can be successfully used for large-scale monitoring of food and beverage marketing exposures.

### Conclusion

The FoodMATS-Youth application had high usability and acceptability among 13 to 17-year-olds to capture food and beverage marketing messages observed within their daily lives. Participants found it easy to use and experienced little to no issues with its functionality. The results from this study demonstrate the opportunity to use the FoodMATS-Youth to fill gaps in evidence on food marketing across settings and show its potential to contribute to food marketing monitoring efforts in Canada and internationally.

## Supplementary material

10.2196/79306 Multimedia Appendix 1 Focus group guide.

## Abbreviations

FoodMATS-Youth: Food and Beverage Marketing Assessment Tool for Settings and Youth
GrabFM: Grab Food Marketing
QUAL: Qualitative
QUAN: Quantitative
