# Supplementary material for: Exploring the Usability and Acceptability of the FoodMATS-Youth App for Monitoring Food Marketing Exposures: Mixed Methods Study
Source: JMIR Form Res. 2025 Dec 9;9:e79306. doi: 10.2196/79306 (PMC12688025; doi:10.2196/79306)
Supplement: Multimedia Appendix 1 [file formative-v9-e79306-s001.docx]

Briefing

Hello everyone, my name is Idris, and I am conducting a study that tests the use of a mobile app to capture food marketing exposures youth face daily. You all have used this app already, and I am interested in learning about your experience using the app and your views on the photos you took. This information will help us understand how the app functioned, the problems you faced, the things you loved, and the things necessary to improve the app. Your participation will help us see this app from a youth perspective.

This focus group will last about one hour, and you can move around. If you need to use the bathroom, you can. This discussion will also be recorded to sufficiently capture your opinions and ideas. Everything said here is confidential, and we will not include your names in anything you say. The audio recording will also be destroyed after transcribing, and no identifying information will be included.

You may choose not to answer any question and withdraw from this focus group whenever you want. There are also no right or wrong answers, and you can have a different experience with the app than others in the group. We want everyone to participate freely, so we are asking you to respect each other’s confidentiality and not share anything said outside this group. Please mute your cell phones so we do not get distracted.

If you have any questions now or later, feel free to ask me. Are there any questions before we get started? If it is fine by everyone, I will start recording and begin the focus group.

**Questions**

1. Tell me your first opinion about the app when you started using it?

Probe: Did that change over the course of the study?

1. You used this app for three days. What was it like to add the app use to your daily activities?
2. Tell me how you felt when you saw some ads outside and had to use the mobile app in public?

Probe: How was using the app at home different from using it outside?

1. What parts of the app did you love the most?

Probe: Why do you love those parts?

1. What were some things you often observed about the ads that the app did not account for?

Probe: Were any locations unclear?

Probe: Are there any other locations you think we should add?

1. Tell me about the problems you encountered when using the mobile app? (if any)
2. How did you feel when you received random notifications and prompts to record the ads you saw?

Probe: When will you have preferred to receive prompts and notifications?

1. What do you think we should change about this app?

Probe: What new features do you think we should add?

1. Looking at the photos you took and what you saw, what marketing techniques do you think these food brands use to appeal to youth?

Probe: What marketing techniques particularly appealed to you?

1. Tell me about a time when you saw a food or beverage ad that made you feel like, "I want that!" or “I would love to have that!”
2. How do you think food ads influence you?

Probe: What of your friends, how does it influence them?

1. Tell me about instances where you saw the same brand in different settings/locations.
2. Were there any similarities between the food marketing ads you saw? Were there any differences between the food marketing ads you saw?

Anything else?

Debriefing

This is the end of our focus group, and I will now stop recording. Thank you all for coming and for your participation. If you have anything else you would like to share, please feel free to do so. Please get in touch with me if you have any additional comments, questions, or concerns after leaving.
